# Antimony resistance mechanism in genetically different clinical isolates of Indian Kala-azar patients

**DOI:** 10.3389/fcimb.2022.1021464

**Published:** 2022-11-02

**Authors:** Supriya Khanra, Shantanabha Das, Nibedeeta Rani Sarraf, Sanchita Datta, Anjan Kumar Das, Madhumita Manna, Syamal Roy

**Affiliations:** ^1^ Department of Zoology, Barasat Government College, Kolkata, India; ^2^ Department of Zoology, Diamond Harbour Women’s University, Sarisha, West Bengal, India; ^3^ Department of Medicine, Calcutta National Medical College, Kolkata, India; ^4^ Department of Infectious Diseases and Immunology, Indian Institute of Chemical Biology, Kolkata, India

**Keywords:** drug resistance, Indian Kala-azar, clinical isolates, *Leishmania donovani*, *L. tropica*

## Abstract

The central theme of this enterprise is to find common features, if any, displayed by genetically different antimony (Sb)-resistant viscerotropic *Leishmania* parasites to impart Sb resistance. In a limited number of clinical isolates (*n* = 3), we studied the breadth of variation in the following dimensions: (a) intracellular thiol content, (b) cell surface expression of glycan having N-acetyl-D-galactosaminyl residue as the terminal sugar, and (c) gene expression of thiol-synthesizing enzymes (CBS, MST, gamma-GCS, ODC, and TR), antimony-reducing enzymes (TDR and ACR2), and antimonial transporter genes (AQP1, MRPA, and PRP1). One of the isolates, T5, that was genotypically characterized as *Leishmania tropica*, caused Indian Kala-azar and was phenotypically Sb resistant (T5-LT-SSG-R), while the other two were *Leishmania donovani*, out of which one isolate, AG83, is antimony sensitive (AG83-LD-SSG-S) and the other isolate, T8, is Sb resistant (T8-LD-SSG-R). Our study showed that the Sb-resistant parasites, regardless of their genotype, showed significantly higher intracellular thiol compared with Sb-sensitive AG83-LD-SSG-S. Seemingly, T5-LT-SSG-R showed about 1.9-fold higher thiol content compared with T8-LD-SSG-R which essentially mirrored cell surface N-acetyl-D-galactosaminyl expression. Except TR, the expression of the remaining thiol-synthesizing genes was significantly higher in T8-LD-SSG-R and T5-LT-SSG-R than the sensitive one, and between the Sb-resistant parasites, the latter showed a significantly higher expression. Furthermore, the genes for Sb-reducing enzymes increased significantly in resistant parasites regardless of genotype compared with the sensitive one, and between two resistant parasites, there was hardly any difference in expression. Out of three antimony transporters, AQP1 was decreased with the concurrent increase in MRPA and PRP1 in resistant isolates when compared with the sensitive counterpart. Interestingly, no difference in expression of the above-mentioned transporters was noted between two Sb-resistant isolates. The enduring image that resonated from our study is that the genetically diverse Sb-resistant parasites showed enhanced thiol-synthesizing and antimony transporter gene expression than the sensitive counterpart to confer a resistant phenotype.

## Introduction

Drug resistance is a serious problem in controlling any kind of infection, be it viral, bacterial, fungal, or protozoan or any other parasitic infection (WHO, 2021). Leishmaniases are no exception. Leishmaniases are neglected tropical diseases that have diversified epidemiology, pathogenesis, and clinical symptoms. Among all leishmaniases disease manifestations, mainly cutaneous, mucocutaneous, and visceral form, visceral leishmaniasis (VL) or Kala-azar (KA) is the most severe type if left untreated (Sundar, 2001). Approximately 20 million people are infected with leishmaniases worldwide (Bhargava and Singh, 2012). KA is endemic in the Indian subcontinent spanning across India, Bangladesh, Brazil, Iran, Nepal, and Sudan (Ostyn et al., 2013). Post-Kala-azar dermal leishmaniasis may develop in about 5–10% of apparently cured KA patients (Gasim et al., 2000). In the Indian subcontinent, KA is caused not only by *L. donovani* (Manna et al., 2005) but also by *L. tropica* (Sacks et al., 1995; Khanra et al., 2012). Among different species belonging to the *Leishmania* genus, the species *L. donovani* and *L tropica* show an anthroponotic nature of transmission (Steverding, 2017). This anthroponotic mode of transmission facilitates the emergence of drug-resistant isolates in these species, unlike the other zoonotic *Leishmania* spp. (ZVL and ZCL) in the Old World (Bamorovat et al., 2021). The main strategies to control the diseases associated with *L. donovani* and *L. tropica* are by developing effective control measures for primary human host infection (Bamorovat et al., 2021), early case detection (Oliaee et al., 2018), and prompt effective treatment (Singh and Sundar, 2015). According to the WHO, all cases must be treated because humans are reservoirs for transmitting the disease to others *via* sandfly vectors (WHO, 2022).

The emergence and progression of resistance of parasites to the commonly used antimonials and other drugs (Purkait et al., 2011; Khanra et al., 2017) made the control strategy inadequate (Capela et al., 2019). Researchers in the field emphasized that the emergence of antimonial resistance in *Leishmania* parasites may be related to sodium stibogluconate (SSG) treatment failure in the Indian state of Bihar (Rijal et al., 2003; Dube et al., 2005), and in the year 2000, the percentage of treatment failure cases had rose up to 60–70% in that region (Sundar et al., 2000). Amphotericin B, pentamidine, paromomycin, and miltefosine are other alternative drugs used for the disease, and they have their own limitations (Sundar, 2001; Moore and Lockwood, 2010).

The mechanism of parasite resistance towards the drug SSG is primarily *via* reduced drug concentration within the parasite, either by a low level of drug uptake or by a high level of drug efflux, and there are some other possible resistance mechanisms. These are related to the inefficiency in the conversion of pentavalent to trivalent antimony and the high level of trivalent antimony detoxification (Decuypere et al., 2005). All of these events related to SSG resistance are governed either by the thiol-metabolizing genes or by antimony transporter genes (Croft et al., 2006). Several types of ATP binding cassette (ABC) transporters are reported to be related to multi-drug resistance (MDR), and the MDR-related protein named multidrug-resistant protein A has been amplified in different *Leishmania* spp. in response to drugs *in vitro* (El Fadili et al., 2005; Coelho et al., 2007). A previous investigation demonstrated that thiol metabolism plays an essential role to preserve the intracellular reducing environment, and as a result, parasites can avoid the harm caused by oxidative stress within the macrophages (Wyllie et al., 2004). Altered levels of thiol have also been reported in Indian field isolates (Mandal et al., 2007; Singh et al., 2014).

The primary aim of our study was to investigate the drug resistance mechanism of genotypically diverse sodium stibogluconate-resistant (SSG-R) clinical isolates (T5 and T8) with respect to a sodium stibogluconate-sensitive (SSG-S) reference isolate (AG83). By the word “genotypically diverse field isolates”, we meant both T8 and T5 are SSG-R and collected from confirmed Kala-azar patients, but their species status is completely different. T8 is the classical *L. donovani* isolate, while T5 belongs to *L. tropica* species (Khanra et al., 2016). As stated above, the third isolate (AG83) taken here for comparison is *L. donovani* and SSG-S. The purpose is to examine the relationship between the evolution of molecular mechanism and genetic background in the heterogeneous field isolates.

To our knowledge, this is the first such kind of report in which the antimony resistance mechanism of two resistant isolates belonging to two different *Leishmania* species causing the same disease condition has been evaluated, and the results were expressed in comparison with antimony-sensitive *L. donovani*.

## Materials and methods

### Ethics statements

Bone marrow aspirates collected from Kala-azar patients were approved by the Ethical Committee of the Calcutta National Medical College, Kolkata. Patient details and the Human Ethics Clearance related statements have already been published previously (Khanra et al., 2012; Sarraf et al., 2021).

### Animal ethics statements

BALB/c mice (*Mus musculus*) and hamsters (*Mesocricetus auratus*) were maintained and bred under pathogen-free conditions. Animal use was approved by the Institutional Animal Ethics Committees of the Indian Institute of Chemical Biology, Kolkata, India. Animal infections to maintain the parasites *in vivo* were performed according to the National Regulatory Guidelines issued by the Committee for the Purpose of Supervision of Experiments on Animals (IICB/AEC-15-2008, 10.06.2008), Ministry of Environment and Forest, Government of India.

### *Leishmania* parasite culture

The present study has been carried out with three (*n* = 3) clinical isolates of KA patients, from which one is the *L. donovani* isolate AG83 (MHOM/IN/1983/AG83), which is used as a reference strain for the present study. The other two named as T5 (MHOM/IN/2010/T5) and T8 (MHOM/IN/2012/T8) were collected in the year between 2010 and 2012 from the confirmed VL patients, and the respective patients’ details were reported previously (Khanra, 2015; Khanra et al., 2016; Sarraf et al., 2021) and are delineated in **Supplementary Table S1**. T5 and T8 parasites were isolated from bone marrow aspirates. Briefly, the sternum or iliac crest was punctured by a special type of needle, and marrow was aspirated using a syringe containing an anticoagulant, such as heparin or EDTA. Only two to three drops were added to each culture tube containing Schneider’s *Drosophila* medium with 20% fetal calf serum (FCS). The *Leishmania* amastigotes were confirmed by Giemsa staining, and after the transformation of amastigotes to promastigotes, the parasites were maintained in M199 medium (St. Louis, MO, USA) with 10% fetal bovine serum. The other one is the *L. donovani* isolate AG83 (MHOM/IN/1983/AG83), which was collected in the year 1983, maintained in M199 medium with 10% FCS, and used here as a reference strain. All the isolates were maintained in infected mice/hamster, and heavily infected animals were sacrificed, with their infected organs cultured to get large numbers of promastigotes. The promastigotes (1 × 10^6^) were kept frozen in the vials. Depending on our need the frozen vial was thawed and propagated one or two cycles in M199 containing 10% FCS to conduct experiments. Our earlier report based on PCR-RFLP and SSCP analysis revealed the genotyping status of T8 and T5, where T5 was found to be *L. tropica* and T8 was found to be *L. donovani*, and our *in vitro* amastigote–macrophage model data shows that both of the parasites are less sensitive towards the drug SSG, with EC_50_ values of 45.17 ± 3.55 and 18.77 ± 4.84 µg/ml for T5 and T8, respectively (Khanra et al., 2016). The SSG unresponsiveness of the T8 parasite was further supported by whole genome analysis (Sarraf et al., 2021). On the other hand, the reference AG83 parasite has been reported as SSG-sensitive parasite (Mukhopadhyay et al., 2011; Palit et al., 2012). Hence, we named T5 and T8 as T5-LT-SSG-R and T8-LD-SSG-R, respectively, and AG83 as AG83-LD-SSG-S in the present study.

### Measurement of non-protein thiol content in *Leishmania* parasites

The promastigotes (2 × 10^6^ each/ml) were washed with phosphate-buffered saline (PBS) thrice and then incubated for 20 min at 37°C with the addition of 5 μM 5-chloromethylfluorescein-diacetate (Molecular Probes, CA, USA) (Sarkar et al., 2009). The labeled promastigotes were subjected to BDFACSAria II cell sorter for cytometric measurement, and the analyses were performed using FACSDIVA software (BD Biosciences, San Jose, CA, USA).

### Measurement of surface sugar residue in *Leishmania* parasites

The expression of surface glycan in promastigotes was carried out as described previously (Mukhopadhyay et al., 2011). Briefly, the promastigotes were washed three times with 1X sterile PBS and incubated with FITC-labeled horsegram (*Dolichos biflorus*) lectin at a dilution of 1:50 for 30 min in FACS buffer. After labeling, flow cytometric measurement of the FITC-labeled promastigotes was performed using BDFACSAria II cell sorter. The mean fluorescence intensity was analyzed by FACSDIVA software (BD Biosciences, San Jose, CA, USA) to determine the expression of surface sugar.

### RNA isolation, cDNA synthesis, and semi-quantitative reverse transcription-PCR

RNA extractions were carried out from all of the harvested promastigotes by disrupting in Trizol solution (Khanra et al., 2017). For the synthesis of cDNA, the total RNA (2 μg/reaction) was first incubated with gene-specific reverse primers, heated at 75°C for 10 min, and immediately kept in ice. A common master mixture (2 μl of 0.1 M DTT, 5 μl of 5X FS buffer, 2 μl of 10 mM dNTP, and 0.5 μl of mouse Moloney leukemia virus reverse transcriptase) was added to the ice-cold RNA mixture and then incubated at 37°C for 90 min, followed by 70°C for 15 min (Banerjee et al., 2009). The freshly prepared cDNA was then amplified in a reaction mixture containing 0.5 μl of cDNA with 1 μl 10 mM dNTP, 1.5 μl 50 mM MgCl_2_, and 0.5 μl Taq polymerase as well as gene-specific primers (**Supplementary Table S2**). Amplification reactions were performed with an Applied Biosystems thermocycler. The cycling conditions for genes of interest were 5 min at 95°C, followed by 30 cycles of denaturation at 95°C for 30 s, annealing at 55–60°C for 3 0s, and extension at 72°C for 30 s. The identities of the PCR-amplified respective gene products were checked by agarose gel electrophoresis. Identical aliquots were investigated in parallel without reverse transcriptase to eliminate the presence of residual genomic DNA contamination in PCR amplification preparations. Densitometry analyses were performed using ImageJ software (v1.41o) (Mukherjee et al., 2012). For the densitometric calculations, the same band area was used to determine the band intensity and normalized for GAPDH. GAPDH was used as the internal control gene.

### Statistical analyses for the *in vitro* study

Each experiment was performed at least three times. The results are expressed as mean ± SD. GraphPad prism software was used to perform Student’s *t*-test for significance analysis, and a *P*-value <0.05 was considered to be significant. The correlation between the EC_50_ and other parameters was measured by the Spearman rank correlation coefficient and represented by R (Khanra et al., 2017).

## Results

### Intracellular thiol content

**Figure 1:**
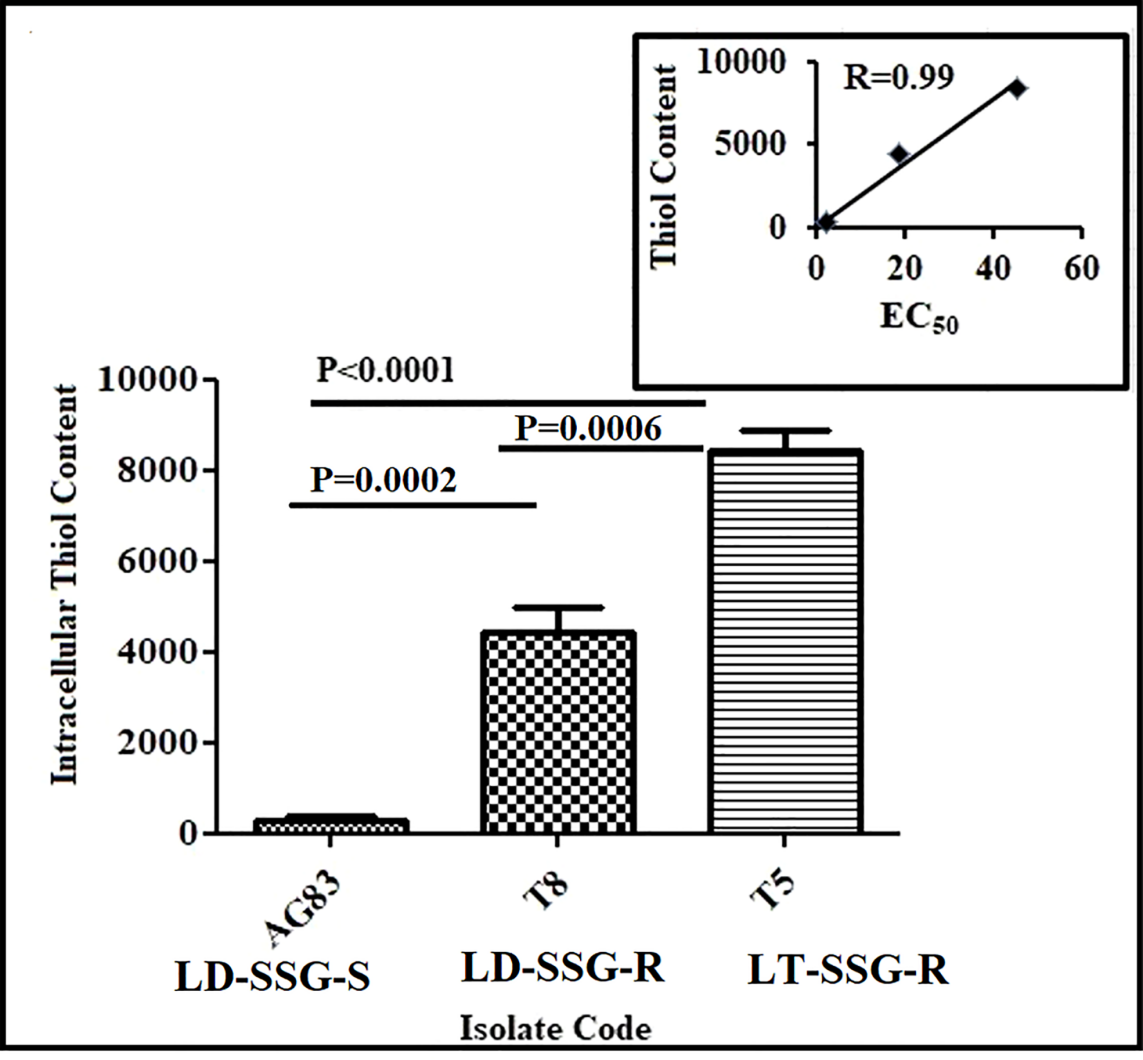
Analysis of thiol content in SSG-S (AG83-LD-SSG-S) and SSG-R (T5-LT-SSG-R and T8-LD-SSG-R) *Leishmania* promastigotes by flow cytometry. The thiol content was measured using a fluorescence probe 5-chloromethylfluorescein-diacetate and presented in terms of mean fluorescence intensity values. The inset shows the scatter plot representing the correlation between EC_50_ values against sodium stibogluconate and the intracellular thiol content of each isolate.

The thiol content was determined in the promastigotes. It was observed that there was a significantly increased thiol content in resistant parasites than in sensitive parasites. It was about 30-fold (*P* < 0.0001) and 16-fold (*P* = 0.0002) increase in thiol content in Sb-resistant T5-LT-SSG-R and T8-LD-SSG-R, respectively, compared with Sb-sensitive AG83-LD-SSG-S. Between the two genetically diverse parasites, T5-LT-SSG-R showed about 1.9-fold (*P* = 0.0006) higher thiol content than T8-LD-SSG-R. Our data revealed a high correlation (*R* = 0.99) between intracellular thiol content and SSG resistance (**Figure 1**, **Supplementary Figure S1**).

### Expression of terminal N-acetyl-D-galactosaminyl on cell surface glycan

**Figure 2:**
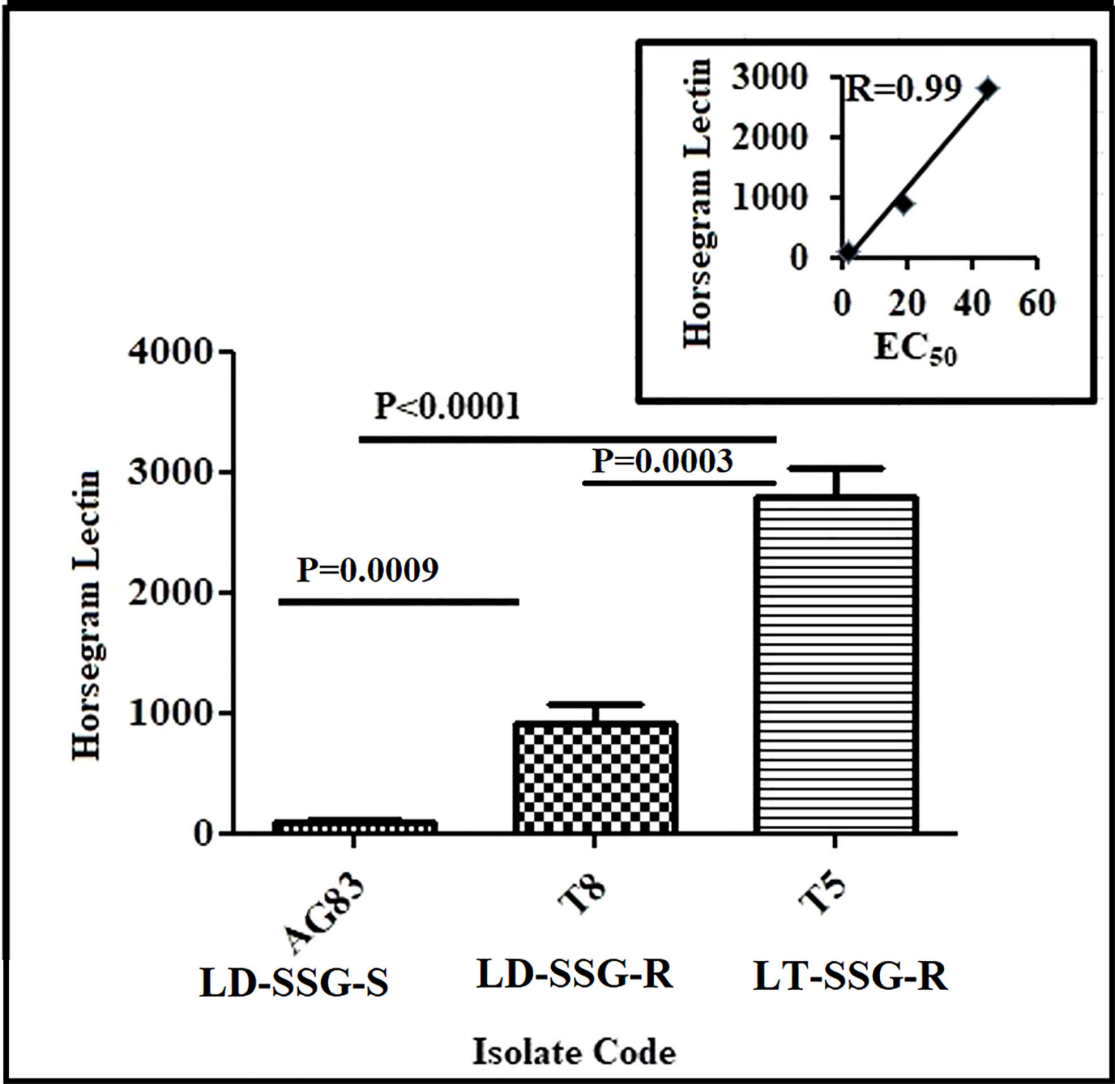
Flow cytometric analysis of the differential expressions of terminal N-acetyl- D-galactosaminyl residue in promastigotes of SSG-R (T5-LT-SSG-R and T8-LD-SSG-R) and SSG-S (AG83-LD-SSG-S) field isolates. The plot shows the binding of FITC-labeled horsegram lectin (*Dolichos biflorus*) in SSG-R and SSG-S isolates. Each scatter plot in the inset represents the correlation between the EC_50_ of the isolates with the presence of surface sugar residue.

The expression of N-acetyl-D-galactosaminyl as the terminal sugar on the cell surface glycan was determined using fluorescence-labeled horse gram (*Dolichos biflorus*) lectin. The binding of lectin was significantly higher in resistant parasites compared with the sensitive counterpart. It was observed that the expression of N-acetyl-D-galactosaminyl as the terminal sugar was about 28-fold (*P* < 0.0001) and ninefold (*P* = 0.0009) higher in Sb-resistant T5-LT-SSG-R and T8-LD-SSG-R, respectively, compared with Sb-sensitive AG83-LD-SSG-S. Between the two genetically diverse parasites, T5-LT-SSG-R showed about threefold (*P* = 0.0003) higher N-acetyl-D-galactosaminyl terminal sugar expression than T8-LD-SSG-R. There was a high correlation (*R* = 0.99) between the expression of N-acetyl-D-galactosaminyl and the SSG sensitivities of the isolates (**Figure 2**, **Supplementary Figure S2**).

### Expression of genes of thiol-synthesizing enzymes

**Figure 3:**
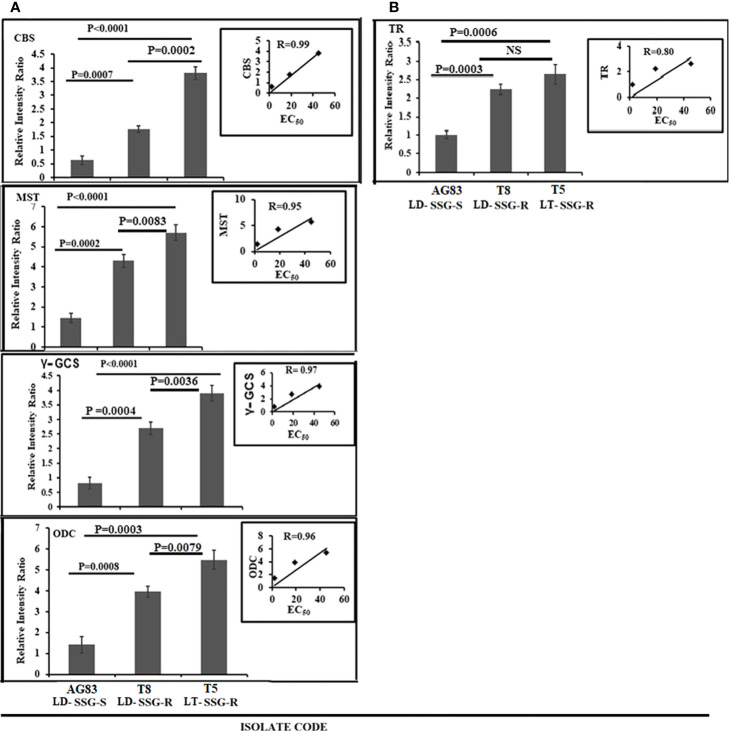
Graphical representation of the densitometric data. **(A)** Expression levels of thiol-metabolizing enzymes CBS, MST, γ-GCS, and ODC were expressed as a ratio of CBS, MST, γ-GCS, and ODC mRNA levels to GAPDH mRNA level, respectively. Data were expressed as the mean ± SD of three independent experiments. Each scatter plot in the inset represents the correlation between the EC_50_ of the isolates against sodium stibogluconate and the respective gene expressions. **(B)** The expression level of another thiol-metabolizing enzyme (TR) was expressed as a ratio of TR mRNA level to GAPDH mRNA level. Data were expressed as the mean ± SD of three independent experiments. Each scatter plot in the inset represents the correlation between the EC_50_ of the isolates against sodium stibogluconate and the respective gene expression. NS, Non-significant.

The expression of cystathionine-ß synthase (CBS), mercaptopyruvate sulfurtransferase (MST), gamma-glutamylcysteine synthase (Ƴ-GCS), ornithine decarboxylase (ODC), and trypanothione reductase (TR) was studied both in Sb-resistant and Sb-sensitive parasites, and the results were expressed as fold increase compared with the sensitive counterpart. The gel pictures of the gene expression profiling experiments are provided in **Supplementary Figure S3**. It was observed that CBS expression was about sixfold higher in T5-LT-SSG-R (*P* < 0.0001) and 2.8-fold higher in T8-LD-SSG-R (*P* = 0.0007) (**Figure 3A**) than in AG83-LD-SSG-S (and the positive correlation value between CBS expressions and EC_50_ of the isolates was determined to be *R* = 0.99). MST expression was 3.9-fold higher in T5-LT-SSG-R (*P* < 0.0001) and 2.9-fold higher in T8-LD-SSG-R (*P* = 0.0002) than in AG83-LD-SSG-S, with a positive correlation value *R* = 0.95 (**Figure 3A**). The other gene Ƴ-GCS was about fivefold over-expressed in T5-LT-SSG-R (*P* < 0.0001) and approximately 3.3-fold over-expressed in T8-LD-SSG-R (*P* = 0.0004) compared with AG83-LD-SSG-S, with a positive correlation between EC_50_ and Ƴ-GCS expression (*R* = 0.97, **Figure 3A**). Similarly, ODC expression was upregulated approximately 3.9-fold in T5-LT-SSG-R (*P* = 0.0003) and 2.8-fold in T8-LD-SSG-R (*P* = 0.0008) compared with that in AG83-LD-SSG-S, with a positive correlation value *R* = 0.96 (**Figure 3A**). TR expression (**Figure 3B**) showed approximately 2.6- and 2.2-fold higher expressions in SSG-R isolates (T5-LT-SSG-R; *P* = 0.0006, T8-LD-SSG-R; *P* = 0.0003, *R* = 0.80) than in the SSG-sensitive counterpart (AG83-LD-SSG-S). These results have also pointed out the enhanced over-expression of those four genes (CBS, Ƴ-GCS, MST, and ODC) except TR in T5-LT-SSG-R (SSG-R *L. tropica*), which ranged from approximately 1.3- to 2.2-fold compared with T8-LD-SSG-R (SSG-R *L. donovani*) (**Figures 3A, B**).

### Expression of genes for antimony reduction

**Figure 4:**
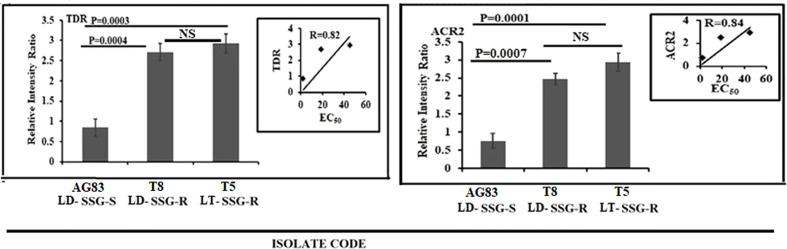
Graphical representation of the densitometric data of the genes responsible for the reduction of pentavalent to trivalent antimony. The expressions of ACR2 and TDR were expressed as the ratio of ACR2 and TDR mRNA levels to GAPDH mRNA level, respectively. Data were expressed as the mean ± SD of three independent experiments. Each scatter plot in the inset represents the correlation between the EC_50_ of the isolates against sodium stibogluconate and the respective gene expression. NS, Non-significant.

The expression level of thiol-dependent reductase (TDR) and arsenate reductase2 (ACR2) was studied and normalized for GAPDH (**Figure 4**). ACR2 expression (**Figure 4**) was approximately 3.8-fold higher (*P* = 0.0001) in T5-LT-SSG-R and 3.2-fold higher (*P* = 0.0007) in T8-LD-SSG-R than in AG83-LD-SSG-S with *R* = 0.84, whereas TDR expression (**Figure 4**) showed around 3.5 fold higher (*P* = 0.0003) expression in T5-LT-SSG-R and 3.2 fold higher (*P* = 0.0004) expression in T8-LD-SSG-R than in AG83-LD-SSG-S with R = 0.82. The respective gel pictures of the gene expression profiling experiments can be found in **Supplementary Figure S3**.

### Expression of genes for antimony transport

**Figure 5:**
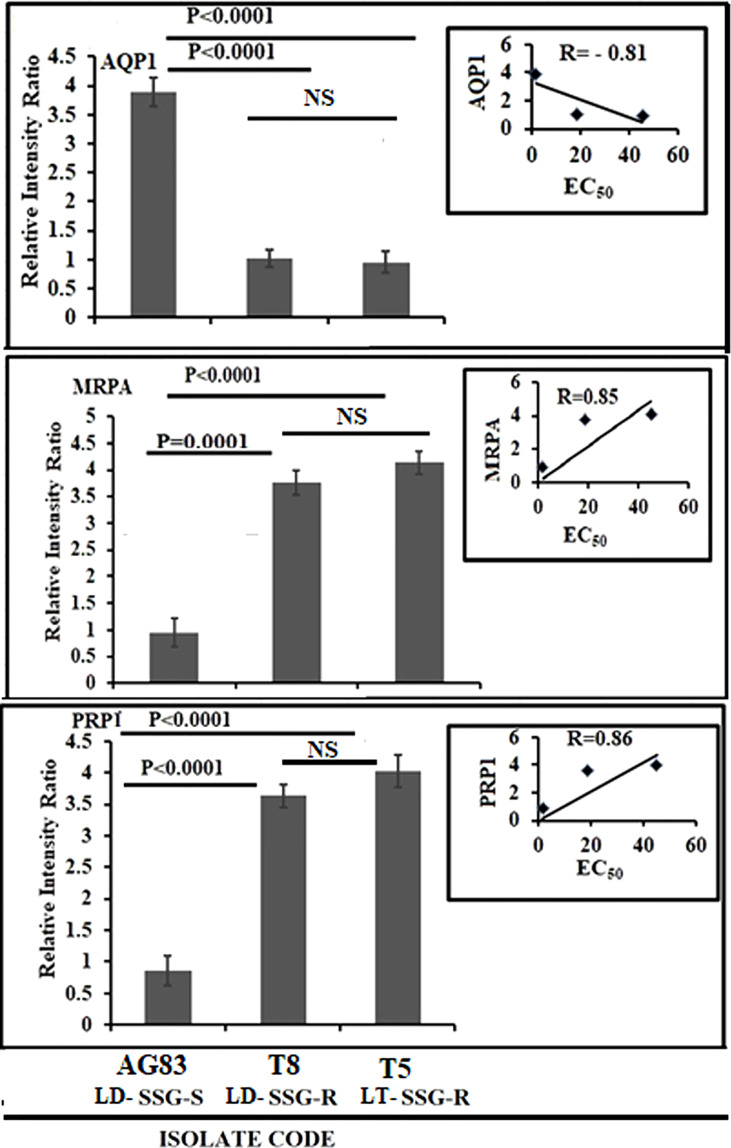
Graphical representation of the densitometric data of sodium stibogluconate (SSG)-transporting enzyme and ABC transporter. The expression level of AQP1 was expressed as a ratio of AQP1 mRNA level to GAPDH mRNA level. Data were expressed as the mean ± SD of three independent experiments. Each scatter plot in the inset represents the correlation between the EC_50_ of the isolates against SSG and the respective gene expression. The expression levels of MRPA and PRP1 were expressed as the ratio of MRPA and PRP1 mRNA levels to GAPDH mRNA level, respectively. Data were expressed as the mean ± SD of three independent experiments. Each scatter plot in the inset represents the correlation between the EC_50_ of the isolates against SSG and the respective gene expression. NS, Non-significant.

The expression levels of genes for antimony transport such as aquaglyceroporin (AQP1), multidrug-resistant protein A (MRPA), and pentamidine resistance protein 1 (PRP1) are significantly altered between SSG-S (AG83-LD-SSG-S) and SSG-R isolates (T5-LT-SSG-R, T8-LD-SSG-R) but not considerably more pronounced between T5-LT-SSG-R and T8-LD-SSG-R relative to the genes stated above. The expression level of AQP1 was found to be upregulated approximately four times (*P* < 0.0001) in AG83-LD-SSG-S parasite compared with that of T5-LT-SSG-R and T8-LD-SSG-R isolates, respectively, and a negative correlation exists between EC_50_ and AQP1 expression (*R* = -0.81) (**Figure 5**; **Supplementary Figure S3**). On the other hand, the MRPA expression (**Figure 5**; **Supplementary Figure S3**) was approximately fourfold (*P* = 0.0001) and 4.4-fold (*P* < 0.0001) over-expressed in T8-LD-SSG-R and T5-LT-SSG-R isolates, respectively, compared with AG83-LD-SSG-S with *R* = 0.85. Similarly, the PRP1 expression was also approximately 4.6- and 4.2-fold upregulated in T5-LT-SSG-R (*P* < 0.0001) and T8-LD-SSG-R (*P* < 0.0001), respectively, with *R* = 0.86 (**Figure 5**; **Supplementary Figure S3**).

## Discussion

An understanding of drug resistance mechanism in respective field isolates is essential because it may offer clues to determine the drug regimen to go for, *e*.*g*., either single drug therapeutics or combination therapy for the treatment of patients. It also helps us to evaluate the progress and extent of resistance in the field (Ashutosh et al., 2007).

Multifactorial determinants are responsible for variations in the drug susceptibility of the field isolates of KA (Croft et al., 2006). A divergence in membrane sterol (Goad et al., 1984; Beach et al., 1988) and lipid (Beach et al., 1979) contents has been established to lead to various drug susceptibility profiles. The wide use of SSG in areas of hyper-endemicity may have changed the biochemical composition of these parasite membranes in ways that might affect drug susceptibility (Kumar et al., 2009). An earlier report demonstrated a variety of biochemical and biophysical changes in SSG-resistant *Leishmania* parasites (Mukhopadhyay et al., 2011) and also delineated the association between the expression level of terminal glycoconjugates and IL-10 induction (Mukherjee et al., 2013).

Our previous investigation based on the *in vitro* amastigote–macrophage model revealed that 46.2% of our studied field isolates were SSG resistant—among them is *L. tropica*, and the others were *L. donovani* (Khanra et al., 2016). A recent report from our group furthermore confirmed the genetic characteristics of some of those SSG-resistant isolates by whole genome analysis (Sarraf et al., 2021). We have taken the SSG R -*L. tropica* (T5-LT-SSG-R; EC_50_ = 45.17 ± 3.55, µg/ml) and one SSG-R *L. donovani* (T8-LD-SSG-R; EC_50_ = 18.77 ± 4.84, µg/ml) clinical isolate along with one SSG-S *L. donovani* (AG83-LD-SSG-S; EC_50_ = 1.87 ± 0.07, µg/ml) isolate for our present study to investigate the relationship between the evolution of molecular mechanism and SSG-resistant phenotype in genetically heterogeneous field isolates.

*Leishmania* produce huge, glycosylated proteins and proteoglycans that have a significant role in parasite virulence (Merida-de-Barros et al., 2018). It is demonstrated that the metacyclogenesis in *Leishmania* parasites is connected with alterations of surface glycoconjugates (Monteiro Tínel et al., 2014) that, in the resistant isolates, were found to be upregulated (Vanaerschot et al., 2010). We have observed significantly higher terminal glycan levels in T5-LT-SSG-R and T8-LD-SSG-R, respectively, than in AG83-LD-SSG-S.

It is documented that the thiol content in the SSG-R isolate is higher than that of the SSG-S isolate (Singh et al., 2014), and our results are offering credence to such a notion. The present study also revealed a very high correlation coefficient (*r* = 0.99) between SSG resistance and intracellular non-protein thiol content in the isolates studied here. Interestingly, our data revealed that the thiol content of the T5-LT-SSG-R isolate is approximately 30.98-fold higher than in AG83-LD-SSG-S and 1.9-fold higher than that of T8-LD-SSG-R isolate. Our data also revealed that T8-LD-SSG-R had approximately 16.30 fold (*P* < 0.0001) higher intracellular thiol content than that of AG83-LD-SSG-S. This observation prompted us to search the potential association between the intracellular thiol content and the expression of genes of thiol-metabolizing enzymes.

Our study demonstrated the over-expression of AQP1 in AG83-LD-SSG-S isolate with respect to T5-LT-SSG-R and T8-LD-SSG-R isolates. AQP1 facilitates SSG uptake into the cell (Haldar et al., 2011; Decuypere et al., 2012). The rest of the genes that are responsible for thiol metabolism and antimony transport were all significantly upregulated in all the SSG-R isolates (T5-LT-SSG-R and T8-LD-SSG-R). Among all of the genes studied, the four genes (CBS, Ƴ-GCS, MST, and ODC) showed a considerable over-expression in SSG-R *L. tropica* isolate (T5-LT-SSG-R) whose range of over-expression varied from 3.8- to 6.1-fold compared with SSG-S *L. donovani* isolate (AG83-LD-SSG-S), whereas it was 1.3- to 2.2-fold higher in the other SSG-R isolate (T8-LD-SSG-R) than in the sensitive counterpart. It has been reported earlier that the pronounced expressions of ODC and Ƴ-GCS in drug-resistant parasites help in the production of trypanothione precursors such as glutathione and spermidine (Grondin et al., 1997; Haimeur et al., 1999) and are also related to an increased thiol level in the antimony-resistant parasites (Mukherjee et al., 2007; Mittal et al., 2007). The other two genes, MST and CBS, also play key roles in the production of cysteine, the other thiol source of trypanosomatids (Nozaki et al., 2001). At this point, it could be strongly postulated that our SSG-R *L. tropica* isolate would show a stronger SSG-resistant phenotype than SSG-R *L. donovani* isolate due to the pronounced expressions of those four specific thiol-metabolizing genes (CBS, Ƴ-GCS, MST, and ODC). This observation also supported our earlier report that showed the higher EC_50_ value of the *L. tropica* isolate, T5-LT-SSG-R (EC_50_ = 45.17 ± 3.55, µg/ml), towards SSG compared with that of other isolates (Khanra et al., 2016). The rest of the genes studied, such as ACR2 and TDR1, have the ability to catalyze the reduction of anti-leishmanial pentavalent antimony to trivalent antimony, and two other genes (MRPA and PRP1) are reported as ABC transporters (Denton et al., 2004; Wyllie et al., 2004; Decuypere et al., 2012). Our present study revealed that the expressions of those genes (ACR2, TDR, TR, MRPA, and PRP1) are not statistically different between two SSG-resistant isolates, T5-LT-SSG-R and T8-LD-SSG-R, but significantly different from SSG-sensitive isolate AG83-LD-SSG-S, which is in congruence with earlier reports stating that the upregulation of MRPA, PRP1, ACR2, TDR, and TR genes may contribute in conferring resistance to the antimonial drug (Mukhopadhyay et al., 2011; Ponte-Sucre et al., 2017).

Our results showed that thiol-metabolizing genes play a crucial role in determining the antimony resistance phenotype in genetically divergent clinical isolates of Indian Kala-azar.

## Conclusion

We may conclude that the altered levels of expression of thiol-metabolizing genes are responsible for the antimony-resistant phenotype in genetically different resistant isolates causing Indian Kala-azar.

### Limitation and strength of the study

The limitation of the present study is the small sample size (*n* = 3). The strength of the present study is that it depicted the comparative profiles of the antimony resistance mechanism of two recent clinical isolates identified as *L. donovani* (T8-LD-SSG-R) and *L. tropica* (T5-LT-SSG-R), which are both resistant to SSG. The results were compared with their SSG-sensitive counterpart (AG83-LD-SSG-S). To the best of our knowledge, this is the first report in which we showed that the antimony resistance mechanism of two field isolates belonging to two different *Leishmania* species causing the same disease is essentially similar.

## Data availability statement

The original contributions presented in the study are included in the article/**Supplementary Material**. Further inquiries can be directed to the corresponding authors.

## Ethics statement

The bone marrow aspirates collected from Kala-azar patients were approved by the Ethical Committee of the Calcutta National Medical College, Kolkata. Written consents were obtained from every patient and guardian (in the case of minors) prior to the study. The patients/participants provided their written informed consent to participate in this study. The animal study was reviewed and approved by the Committee for the Purpose of Supervision of Experiments on Animals (IICB/AEC-15-2008, 10.06.2008), Ministry of Environment and Forest, Government of India.

## Author contributions

SK, ShD, NS, SaD, and AD have worked under this project. MM and SR have designed the problem and written the manuscript. All authors contributed to the article and approved the submitted version.

## Funding

We sincerely acknowledge the Department of Biotechnology (DBT Twinning Program, BCIL/NER-BPMC/2013), New Delhi, India, and the University Grant Commission (UGC), New Delhi, India [35/57/2009 (SR)] for financial help. We further acknowledge the Council of Scientific and Industrial Research, New Delhi, India, for the fellowship of SK. This work was also supported by the Network Project (Project NWP 0005), J.C. Bose Fellowship (SB/S2/JCB-65/2014), and ICMR Emeritus Fellowship to SR.

## Acknowledgments

We are thankful to the Director of Public Instruction, Higher Education Dept. Govt. of West Bengal, the Principal, Barasat Govt. College, Kolkata, India, and the Director, Indian Institute of Chemical Biology, Kolkata. We thank Mr. Connor Tennant, doctoral student, Wellcome Centre for Integrative Parasitology, Institute of Infection, Immunity and Inflammation, University of Glasgow for reading and correcting the manuscript.

## Conflict of interest

The authors declare that the research was conducted in the absence of any commercial or financial relationships that could be construed as a potential conflict of interest.

## Publisher’s note

All claims expressed in this article are solely those of the authors and do not necessarily represent those of their affiliated organizations, or those of the publisher, the editors and the reviewers. Any product that may be evaluated in this article, or claim that may be made by its manufacturer, is not guaranteed or endorsed by the publisher.

## Supplementary material

Supplementary Figure 1 Flow cytometric analysis of thiol content in *Leishmania*. Analyses of the thiol content in promastigotes of SSG-S *L. donovani* (**A**: AG83-LD-SSG-S), SSG-R *L. donovani* (**B**: T8-LD-SSG-R), and SSG-R *L. tropica* (**C**: T5-LT-SSG-R) were performed with fluorescence probe 5-chloromethylfluorescein-diacetate. Our result denoted that the thiol content has been varied among the SSG-S and SSG-R isolates studied here.

Supplementary Figure 2 Flow cytometric analysis of surface sugar in *Leishmannia* promastigotes. Terminal N-acetyl-D galactosaminyl residues in *Leishmannia* promastigotes were measured by the binding of FITC-labeled horsegram (*Dolichos biflorus*) lectin in SSG-R and SSG-S isolates. Our data revealed the differential expressions of the terminal N-acetyl-D-galactosaminyl residue in the promastigotes of SSG-S *Leishmania donovani* (**A**: AG83-LD-SSG-S) and SSG-R *Leishmania donovani* (**B**: T8-LD-SSG-R) and SSG-R *Leishmania tropica* (**C**: T5-LT-SSG-R).

Supplementary Figure 3 Gel images of the studied gene expressions. GAPDH mRNA expressions: lane 1, AG83-LD-SSG-S; lane 2, T8-LD-SSG-R; lane 3, T5-LT-SSG-R. AQP1 mRNA expressions: lane 1, T8-LD-SSG-R; lane 2, T5-LT-SSG-R; lane 3, AG83-LD-SSG-S. ACR2 mRNA expressions: lane 1, T5-LT-SSG-R; lane 2, T8-LD-SSG-R; lane 3, AG83-LD-SSG-S. CBS mRNA expressions: lane 1, T5-LT-SSG-R; lane 2, T8-LD-SSG-R; lane 3, AG83-LD-SSG-S. γ-GCSmRNA expressions: lane 1, T5-LT-SSG-R; lane 2, T8-LD-SSG-R; lane 3, AG83-LD-SSG-S. MST mRNA expressions: lane 1, AG83-LD-SSG-S; lane 2, T5-LT-SSG-R; lane 3, T8-LD-SSG-R. ODC mRNA expressions: lane 1, T8-LD-SSG-R; lane 2, T5-LT-SSG-R; lane 3, AG83-LD-SSG-S. MRPA mRNA expressions: lane 1, AG83-LD-SSG-S; lane 2, T5-LT-SSG-R; lane 3, T8-LD-SSG-R. PRP1 mRNA expressions: lane 1, T5-LT-SSG-R; lane 2, T8-LD-SSG-R; lane 3, AG83-LD-SSG-S. TDR mRNA expressions: lane 1, AG83-LD-SSG-S; lane 2, T5-LT-SSG-R; lane 3, T8-LD-SSG-R. TR mRNA expressions: lane 1, T8-LD-SSG-R; lane 2, AG83-LD-SSG-S; lane 3, T5-LT-SSG-R.
