# Supplementary figures and images for: Antimony resistance mechanism in genetically different clinical isolates of Indian Kala-azar patients
Source: Front Cell Infect Microbiol. 2022 Nov 2;12:1021464. doi: 10.3389/fcimb.2022.1021464 (PMC9667115; doi:10.3389/fcimb.2022.1021464)

## Slide 1
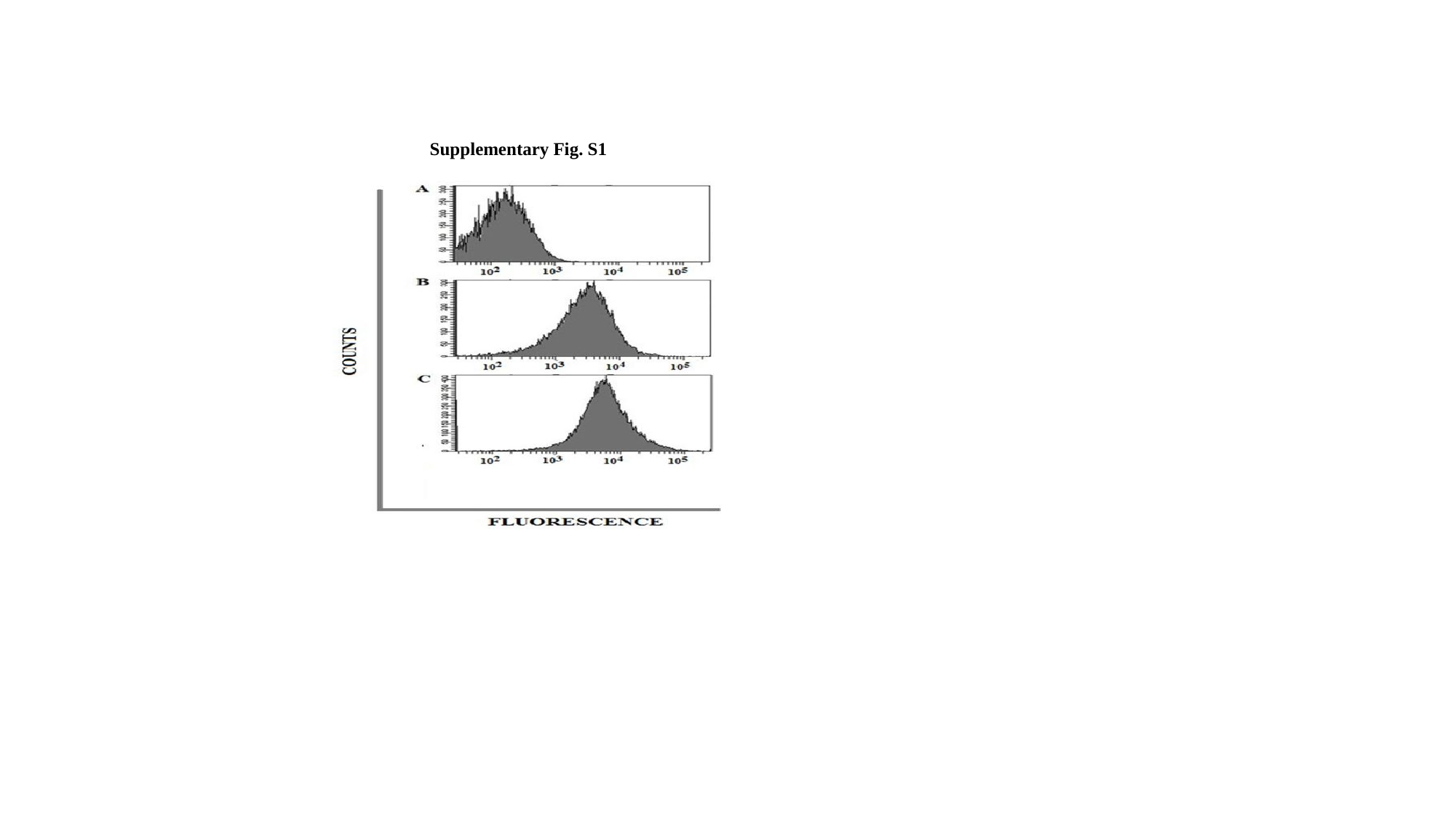

Supplementary Fig. S1

Supplement: Supplementary Figure 1 — Flow cytometric analysis of thiol content in Leishmania. Analyses of the thiol content in promastigotes of SSG-S L. donovani (A: AG83-LD-SSG-S), SSG-R L. donovani (B: T8-LD-SSG-R), and SSG-R L. tropica (C: T5-LT-SSG-R) were performed with fluorescence probe 5-chloromethylfluorescein-diacetate. Our result denoted that the thiol content has been varied among the SSG-S and SSG-R isolates studied here. [file Presentation_1.pptx]

## Slide 1
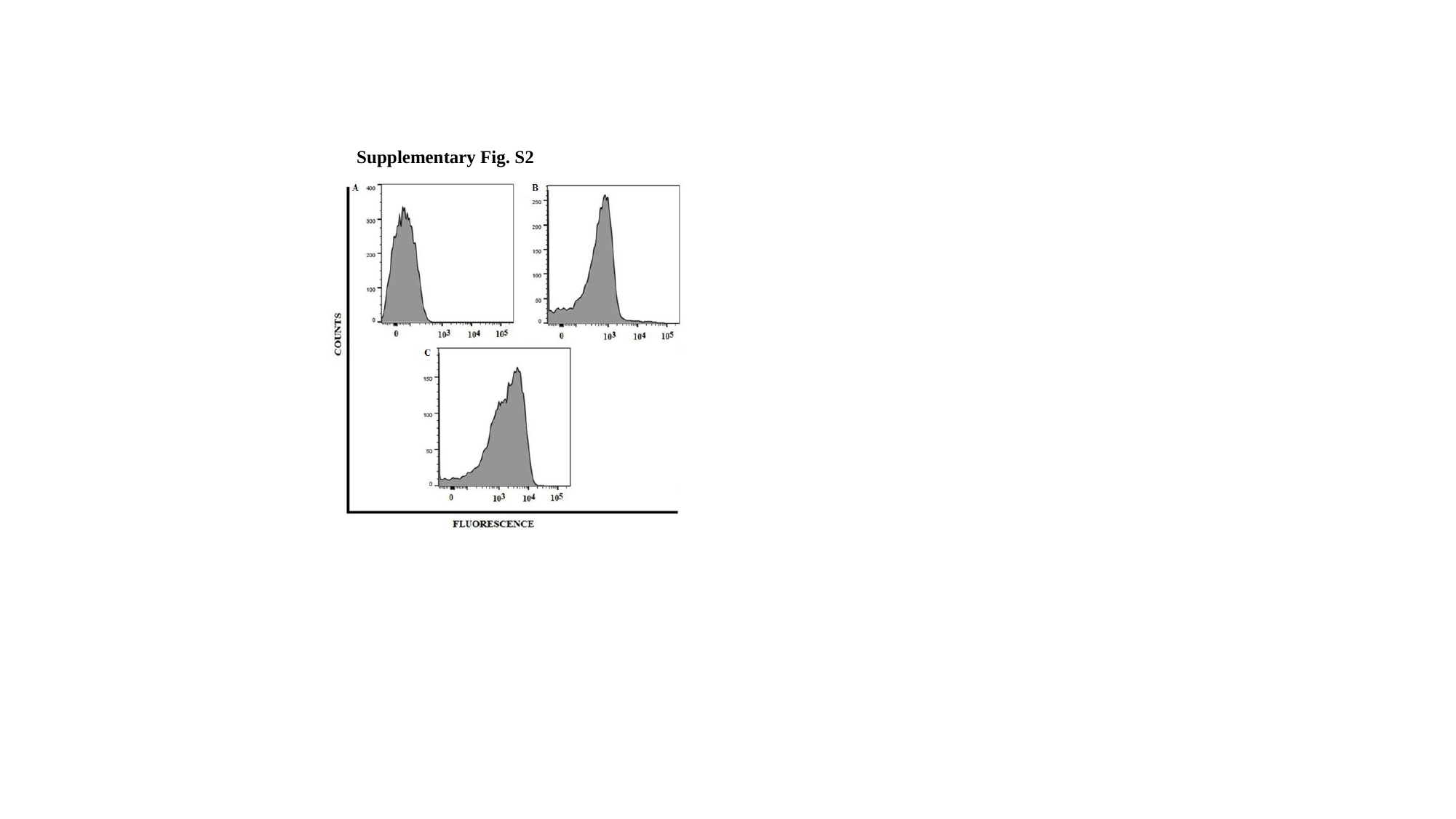

Supplementary Fig. S2

Supplement: Supplementary Figure 2 — Flow cytometric analysis of surface sugar in Leishmannia promastigotes. Terminal N-acetyl-D galactosaminyl residues in Leishmannia promastigotes were measured by the binding of FITC-labeled horsegram (Dolichos biflorus) lectin in SSG-R and SSG-S isolates. Our data revealed the differential expressions of the terminal N-acetyl-D-galactosaminyl residue in the promastigotes of SSG-S Leishmania donovani (A: AG83-LD-SSG-S) and SSG-R Leishmania donovani (B: T8-LD-SSG-R) and SSG-R Leishmania tropica (C: T5-LT-SSG-R). [file Presentation_2.pptx]

## Slide 1
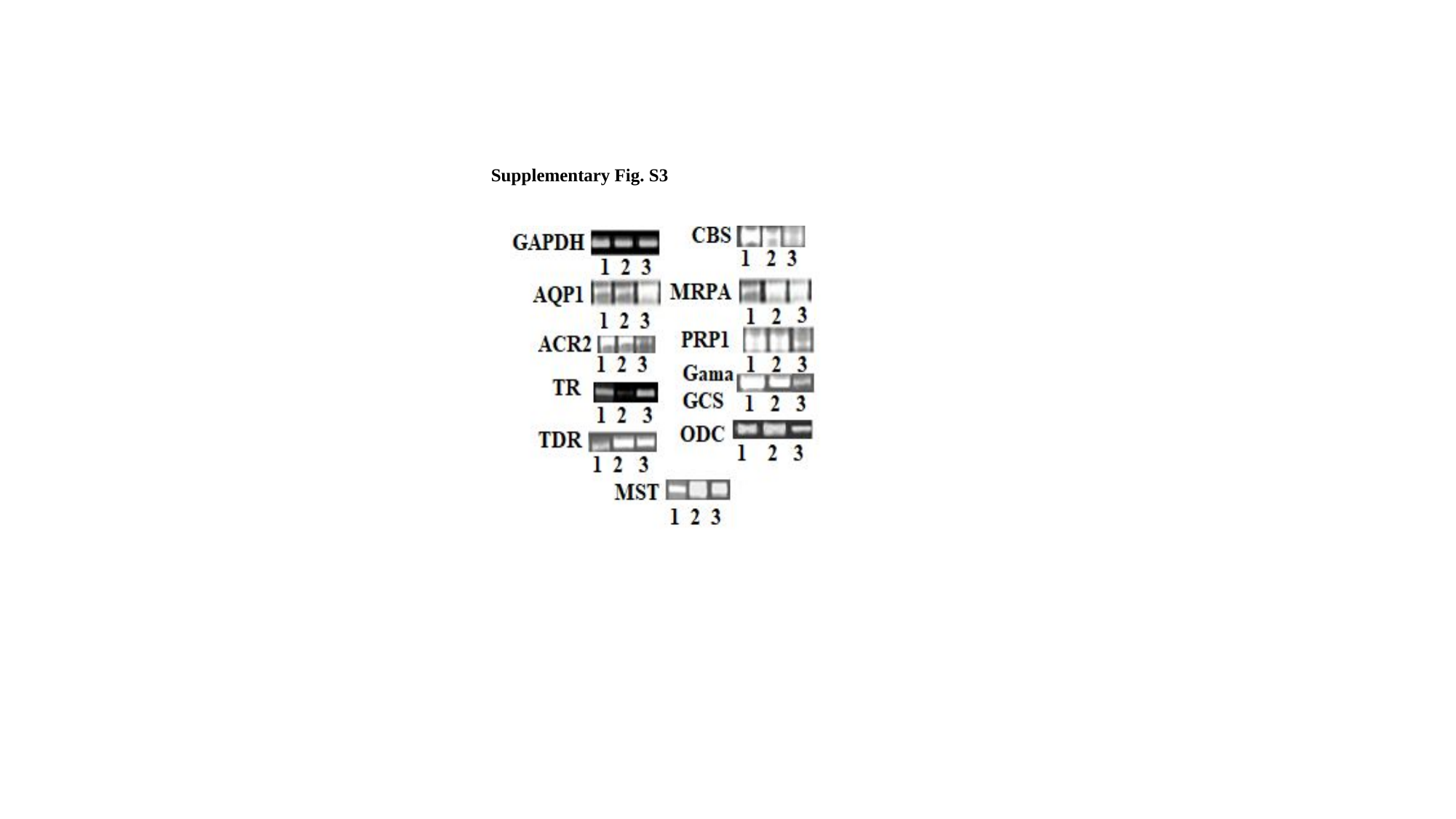

Supplementary Fig. S3

Supplement: Supplementary Figure 3 — Gel images of the studied gene expressions. GAPDH mRNA expressions: lane 1, AG83-LD-SSG-S; lane 2, T8-LD-SSG-R; lane 3, T5-LT-SSG-R. AQP1 mRNA expressions: lane 1, T8-LD-SSG-R; lane 2, T5-LT-SSG-R; lane 3, AG83-LD-SSG-S. ACR2 mRNA expressions: lane 1, T5-LT-SSG-R; lane 2, T8-LD-SSG-R; lane 3, AG83-LD-SSG-S. CBS mRNA expressions: lane 1, T5-LT-SSG-R; lane 2, T8-LD-SSG-R; lane 3, AG83-LD-SSG-S. γ-GCSmRNA expressions: lane 1, T5-LT-SSG-R; lane 2, T8-LD-SSG-R; lane 3, AG83-LD-SSG-S. MST mRNA expressions: lane 1, AG83-LD-SSG-S; lane 2, T5-LT-SSG-R; lane 3, T8-LD-SSG-R. ODC mRNA expressions: lane 1, T8-LD-SSG-R; lane 2, T5-LT-SSG-R; lane 3, AG83-LD-SSG-S. MRPA mRNA expressions: lane 1, AG83-LD-SSG-S; lane 2, T5-LT-SSG-R; lane 3, T8-LD-SSG-R. PRP1 mRNA expressions: lane 1, T5-LT-SSG-R; lane 2, T8-LD-SSG-R; lane 3, AG83-LD-SSG-S. TDR mRNA expressions: lane 1, AG83-LD-SSG-S; lane 2, T5-LT-SSG-R; lane 3, T8-LD-SSG-R. TR mRNA expressions: lane 1, T8-LD-SSG-R; lane 2, AG83-LD-SSG-S; lane 3, T5-LT-SSG-R. [file Presentation_3.pptx]
