# Supplementary material for: Antimony resistance mechanism in genetically different clinical isolates of Indian Kala-azar patients
Source: Front Cell Infect Microbiol. 2022 Nov 2;12:1021464. doi: 10.3389/fcimb.2022.1021464 (PMC9667115; doi:10.3389/fcimb.2022.1021464)
Supplement: Supplementary file 5 [file Table_2.docx]

**Supplementary Table S2 - Studied genes and specific primer sequences.**

| **Studied gene ^a^ Primer sequence (5’-3’)** |
| --- |
| Aquaglyceroporin (AQP1) F-CTGTGTCTTTGGTGCCTTTCC  R-GCCTTTTGGGCGTCGTC  Multidrug resistant protein (MRPA) F-CGAAAGTTGAGCAGGAGAC  R-AATCCCCAAGCAGCCAGAC  Thiol dependent reductase (TDR) F-GTGGCGAGGATGCGAAGG  R-CGGACCAGGAAAGGTAGAATAGC  Gamma-Glutamylcysteine synthase (gamma-GCS) F-TTTGCGTCCTGGTGCCTC  R-TCAATGTTTAGTTGGGGGTC  Trypanothione reductase (TR) F-GGCGAGGTTCTGGGTGTTC  R- GACTCCGATGGTGCTGTGG  Mercaptopyruvate sulfurtransferase (MST) F-GGAGGAGAACCGCCACAAC  R-GCCGCAGGAGAAGACGAAG  Cystathionine-ß-synthase (CBS) F-CGCCGATGTCAACTGGATG  R-GCTCCTTCTTCAGCGTGTCG  Pentamidine resistance protein 1 (PRP1) F-TGATTCCCTTTTTGGGCATTA  R-CGTAGAACTTGAGCAGGAGCAC  Arsenate reductase2 (ACR2) F-GCCCAGTCGCTCATACGG  R-AGAACGCCTCCCACCCAC  Ornithine decarboxilase (ODC) F-ATCCACCTCCAACCCGC  R-TCCGCAACAGCAACAACAG |

^a^ The studied genes responsible for thiol metabolism and SSG transport
